# Subsidence of All-Polyethylene Tibial Components Used in Unicondylar Knee Arthroplasty: A Cohort Study

**DOI:** 10.7759/cureus.39904

**Published:** 2023-06-03

**Authors:** Ehab S Saleh, Sazid Hasan, William A Jiranek

**Affiliations:** 1 Department of Orthopedic Surgery, Oakland University William Beaumont School of Medicine, Rochester, USA; 2 Department of Orthopedic Surgery, Duke University, Durham, USA

**Keywords:** complications, depuy preservation, subsidence, all polyethylene tibial component, unicompartment knee arthroplasty

## Abstract

Introduction: Unicompartmental designs and techniques have been developed to preserve bone stock and minimize soft tissue trauma. Early modern designs and techniques have been introduced with little support in the peer-reviewed literature.

Material and methods: From October 2002 to May 2004, 64 consecutive DePuy Preservation unicondylar knee arthroplasties (UKAs) were performed in 56 patients. Two patients died of unrelated consequences, leaving 62 UKAs for review (55 medial, 7 lateral). All procedures were performed through a quadriceps-sparing approach. All components were cemented, including an all polyethylene tibial component. Clinical and radiographic follow-up data were reviewed and analyzed.

Results: At an average follow-up of 2.5 years, six (11%) of the medial tibial components have subsided. Of these, four had moderate-to-severe pain, one did require a revision to a total knee arthroplasty (TKA), and another did stabilize. An additional two patients continued to have knee pain (one requiring conversion to TKA), leaving a total of 55 UKAs (89%) functioning well at early follow-up. Additional complications have included four deep vein thromboses, three cardiac issues following the index procedure, one surgical site infection, one intraoperative medial femoral condyle fracture, and one reoperation for loose cement fragments.

Conclusion: This study demonstrates a high rate of subsidence for all-polyethylene tibial components used in UKA, resulting in pain and failure of the arthroplasty. Despite the less invasive approach, we found complications that are usually associated with TKA surgery as well as those unique to UKA.

## Introduction

There has always been an interest in performing unicondylar knee arthroplasty (UKA) for the isolated unicompartmental disease of the knee. Articles and evidence from registry data have shown promising mid- to long-term results. Berger et al. reported the results with survivorship free of revisions of 95.7% ± 4.3% at 15 years [1]. Other authors have reported similar results [2-4]. The Swedish Knee Registry shows survivorship at 10 years in the range of 90% for a variety of implant designs.

UKA is appealing in the treatment of isolated diseases of the knee due to its perceived less invasive nature and preservation of normal kinematics in the joint. The advent of less invasive or minimally invasive surgery has made UKA more appealing to the surgeon and the general public because it can be performed in a quadriceps-sparing manner. The procedure is often performed in an outpatient or overnight setting, and when admitted, patients typically have a shorter hospital stay [5]. Furthermore, it can be performed in a relatively bone conserving manner, preserving bone stock for possible future surgeries. All of these factors have ushered in an increased interest in UKA as a legitimate means of dealing with the significant problem of unicompartmental osteoarthritis of the knee. Orthopedic surgeons and implant companies have developed instrumentations, techniques, and implants in order to perform UKA in this manner.

Despite the perceived benefits of performing UKA through a quadriceps-sparing approach with a bone conserving implant, at the time of this study, little data had been presented in support of this philosophy [6]. Hamilton et al. presented their data using this approach and reported a reoperation rate of 11.3% [7].

It is in this light that we have chosen to review the initial experience of one surgeon with the DePuy Preservation UKA.

## Materials and methods

The first 64 consecutive DePuy Preservation UKAs performed by the senior author on 56 patients from October 2002 through May 2004 were reviewed. No other UKAs were performed during this time period. Two patients died of unrelated causes during this time period leaving 62 knees for review, of which 55 were medial and 7 were lateral. All patients received the DePuy Preservation unicondylar arthroplasty implant, which consisted of a cemented all-polyethylene tibial component and a cobalt chrome femoral component. All patients had a diagnosis of osteoarthritis except for one patient with avascular necrosis. All patients had an intact anterior cruciate ligament and had minimal to no osteoarthrosis involving the patellofemoral or contralateral compartment.

A chart review was used to identify the patients. Preoperative and postoperative data, including both radiographic and clinical data, were compiled. Knee Society and function scores were calculated. Preoperative, initial postoperative, and latest follow-up radiographs were examined. Any patients with a follow-up of less than two years were contacted by phone and answered a phone survey to calculate their Knee Society scores. Any patients with poor Knee Society scores or moderate-to-severe pain were brought back for current radiographs and clinical exam. No patients were lost to follow-up.

The average age of the 54 patients was 56 ± 10 years (range: 49-86 years) with 17 males and 37 females. The body mass index (BMI) averaged 29.2 ± 5 (range: 21.1-42.5). The tibial component thickness used included sizes of 7 mm (48), 9.5 mm (13), and 11.5 mm (one) (Table 1). The average preoperative Knee Society score was 52.6 ± 11.2, and the average function score was 51.9 ± 10.5.

**Table 1:** Patient and implant demographics. BMI: body mass index.

Demographics	Numbers
Patients	54 (8 bilateral knees)
Sex	37 Females
	17 Males
Knees	62
Age	56 ± 10 years (range: 49-86 years)
BMI	29.2 ± 5 (21.1-42.5)
Side	55 Medial
	7 Lateral
Tibial thickness	7 mm (48 knees)
	9.5 mm (13 knees)
	11.5 mm (1 knee)
Tibial size	Size 5 (9 knees)
	Size 4 (25 knees)
	Size 3 (27 knees)
	Size 2 (1 knee)
Femoral size	Size 5 (6 knees)
	Size 4 (28 knees)
	Size 3 (25 knees)
	Size 2 (3 knees)

The surgical procedure was performed in a standardized manner through a quadriceps-sparing approach. The incision was made from the superior pole of the patella to 3-4 cm below the joint line. A medial or lateral arthrotomy was performed from the superior pole of the patella to approximately 2 cm below the joint line. The medial collateral ligament was not released. The tibia was cut using an extramedullary guide, making a cut perpendicular to the long axis of the tibia. An attempt was made to match the patient's tibia slope. A conservative bone cut was made in order to remove 2 mm of bone from the tibia. The knee was placed in 90 degrees of flexion, and a 7 mm spacer block was placed. Additional tibial bone was resected if necessary to make a 7 mm spacer block fit. If necessary, a larger spacer block (9.5 mm, 11.5 mm) was placed to fill in the flexion space. The knee was then brought into full extension, and the extension space was balanced to match the flexion space. The femoral finishing guide was placed to optimize femoral tracking on the polyethylene. The tibial component was cemented in place followed by the femoral component using a single patch of cement. The knee was brought into full extension and held until the cement had fully cured.

Postoperatively, all patients were admitted and given standard postoperative care, including 24 hours of intravenous antibiotics, regional versus patient-controlled intravenous analgesia, and deep vein thrombosis prophylaxis with warfarin (goal: international normalized ratio (INR) 1.5-2.5). Patients were allowed to be weight-bearing as tolerated with crutches or a walker on the day of surgery and were placed in a continuous passive motion machine during their hospitalization. The majority of patients were discharged on postoperative day 2. Patients were subsequently seen for follow-up at 10-14 days, six weeks, 12 weeks, and yearly.

## Results

At an average follow-up of 2.5 years (range: 24-43 months), 60 of the UKA remained intact and free from revision. The average Knee Society score was 92 ± 11, and the average function score was 83 ± 20. Six (11%) of the 55 medial tibial components have subsided. Of these, one required revision to a total knee arthroplasty (TKA), four had moderate-to-severe pain with an average Knee Society score of 66 and an average function score of 57, and the sixth patient had stabilized and is functioning well. None of the lateral UKAs subsided. Two additional patients continued to have pain in the knee after their UKA, without subsidence, with one being converted to a TKA at one year due to continued pain. One patient required reoperation with arthroscopy for the removal of loose cement fragments. Fifty-five (89% ) of the 60 UKAs that were not revised to a TKA functioned well at early follow-up.

Radiographic evaluation of the UKA revealed the tibial components were placed in an average of 2.1 degrees of varus (range: 2 degrees of valgus to 7 degrees of varus). The average anatomic axis measured 5.4 degrees of valgus (range: 0-10 degrees). The tibial slope averaged 8 degrees (range: 3-14). Sixteen of the knees were noted to have excess cement posterior to the tibial component on lateral radiographs.

No difference was noted between patients with subsidence of the tibial component and those without subsidence in regard to patient demographics, component position, or limb alignment. Additional complications have included four deep vein thromboses, three cardiac events, one surgical site infection, and one intraoperative medial femoral condyle fracture that required internal fixation (Table 2). 

**Table 2:** Types of complications.

Types of complications	Number of complications
Tibial component subsidence	Six tibial components (one patient needed revision)
Deep vein thrombosis	Four patients
Cardiac complications	Three patients
Continued knee pain without tibial component subsidence	Two patients (one patient needed revision surgery)
Surgical site infection	One patient
Intraoperative fracture	One patient
Loose cemented fragments	One patient required knee arthroscopy
Total complications	18/62 (29%)

## Discussion

The success of UKA and the ability to perform the procedure in a less invasive manner have increased the interest and utilization of the procedure. It is important to report the early results and experience with all designs implanted in a less invasive manner.

Our results demonstrate a high rate of failure (11%) of cemented all-polyethylene tibial components at a 2.5-year follow-up. Hamilton et al. reported similar results with the same prosthesis design, with a revision rate of 4.1% [7]. In contrast to our findings, they noted four cases of isolated femoral loosening, two cases of tibial loosening, and two cases of both tibial and femoral component loosening. They did not comment on or report their results for patients who may have had problems clinically or radiographically but otherwise had intact components.

We could not identify specific factors that contributed to failure in our patients. Previous authors have noted that certain factors may lead to failure of UKA, either contributing to advanced wear of the polyethylene or advancement of arthrosis in other compartments of the knee [8-10]. Collier et al. reported that risk factors for revision of UKA with a follow-up of nine years included patient age, polyethylene shelf age, tibial component thickness, decrease of medial tibial plateau varus, and hip-knee-ankle angle [11].

We propose that there may be something implant-specific about this all-polyethylene tibial component design that causes early failure. Factors that could play a role include a relatively medially placed tibial keel and a lack of metal backing. In contrast, other authors have shown there to be no differences in early results whether using an all-polyethylene or a metal-backed tray with a different implant system [12].

We also noted a fair number of other complications associated with UKA. This included an overall complication rate of 29%. Prior records on the success of UKA have stated little on their complication rate other than those that were surgical in nature. Despite the ability to perform the procedure in a less traumatic manner, patients had the typical complications seen with reconstructive surgery of the lower extremity. 7% of our patients suffered a deep vein thrombosis that required prolonged anticoagulation. Although only one of our patients required a reoperation for loose cement fragments, we noted that many patients had rather prominent cement protruding behind the tibial tray on lateral radiographs. These patients may be at risk for symptomatic loose bodies requiring reoperation. One advantage of a modular tibial metal-backed tray in this setting is the ability to assess for and remove excess cement from around the tibial tray.

Jain et al. reported their experience with 72 patients using the same implant. No fractures occurred in their series, which the authors attributed to modifying the pin configuration used to secure the tibia cutting block (using one or two pins and not using the vertical cutting guide pin). Two revisions to a TKA occurred in their series, one for developing patellofemoral osteoarthritis and the other for continued pain and recurrent knee effusions and suspected underlying rheumatoid arthritis. Three additional patients had a reoperation without the need to revise the prosthesis; one of those reoperations was the arthroscopic removal of loose cement from the posterior knee, which was attributed to limited exposure during the index procedure [13]. 

Scott et al. reported their 10-year experience using the same implant on 97 patients. They reported a high rate of early failure between two and five years, predominantly due to unexplained pain and tibial-sided failure. Their 10-year survival for this UKA was found to be 85.5%. Unexplained pain was the commonest mode of failure (35%), followed by osteoarthritis progression (29%) and tibial subsidence/loosening (24%) [14].

Bhattacharya et al. reported on the survivorship of 91 UKAs using the Preservation DePuy UK (fixed bearing, with an all-polyethylene tibial component), and for comparison, they reviewed the survivorship of 49 mobile-bearing UKAs (Oxford UKA, Biomet UK Ltd, Bridgend, United Kingdom). They found a higher incidence of revision of the fixed-bearing design using an all-polyethylene tibial component compared to the mobile-bearing design [15].

In a randomized study, Hutt et al. evaluated the outcome of a single design of a fixed-bearing UKA (Accuris UKA, Smith and Nephew, London, United Kingdom) with either a cemented all-polyethylene or a metal-backed modular tibial component. They concluded that the all-polyethylene design of fixed-bearing UKA had unsatisfactory results with significantly higher rates of failure before 10 years when compared with the fixed-bearing metal-back components [16]. 

On the other hand, Plate et al. concluded that although an all-polyethylene tibial component for a UKA is technically challenging, it does have many advantages including bone preservation, restoration of normal knee kinematics, maintenance of capsular structures, improving proprioception, and allowing for higher activity levels. He also added that robot-assisted techniques will improve component positioning and may lead to more favorable outcomes [17]. 

## Conclusions

Although UKA is a viable option for isolated unicompartmental arthritis of the knee, it is important to note that it has a side effect profile similar to that of a TKA. The continued use of an all-polyethylene tibial component using this implant system is brought into question. Using an all-polyethylene tibial component allows for more bone preservation and an easier revision surgery in the future if needed, but the disadvantages include high rates of tibial component subsidence, and limited visualization of the posterior tibia cement mantel. The use of robotic technique can be of help.
